# The first reported case of *Balamuthia* amoebic encephalitis initially presenting as anti-NMDAR encephalitis

**DOI:** 10.3389/fmed.2026.1929027

**Published:** 2026-08-28

**Authors:** Shaoqing Yang, Zhijie Fan, Rui Wu, Lirong Duan, Jingjie Sun

**Affiliations:** 1 The Second Hospital & Clinical Medical School, Lanzhou University, Lanzhou, China; 2 Department of Neurology, The Second Hospital & Clinical Medical School, Lanzhou University, Lanzhou, China

**Keywords:** anti-NMDAR encephalitis, *Balamuthia* amebic encephalitis, *Balamuthia mandrillaris*, magnetic resonance imaging, metagenomic next-generation sequencing

## Abstract

Balamuthia mandrillaris (B. mandrillaris) is a highly pathogenic free-living amoeba that can invade the central nervous system and cause *Balamuthia* amoebic encephalitis (BAE). BAE is associated with an extremely high mortality rate, and no treatment regimen with well-established efficacy is currently available. We report a case of BAE in a 63-year-old man who was initially diagnosed with anti-*N*-methyl-D-aspartate receptor (NMDAR) encephalitis. Because the patient initially exhibited no overt signs of infection and metagenomic next-generation sequencing (mNGS) failed to identify any pathogens in the cerebrospinal fluid (CSF), the presence of frequent seizures and anti-NMDAR antibodies in the CSF initially led the treating clinicians to a diagnosis of anti-NMDAR encephalitis. As the disease progressed, multiple intracranial lesions developed. Repeat mNGS subsequently detected *B. mandrillaris* in the CSF, thereby establishing the diagnosis of BAE. Despite combination antimicrobial therapy, the patient’s condition failed to improve, and he ultimately died. This case underscores the importance of maintaining a high index of suspicion for amoebic infection in patients with encephalitis, as *B. mandrillaris* infection may elicit autoimmune responses early in the disease course and mimic autoimmune or other noninfectious forms of encephalitis.

## Introduction

*Balamuthia mandrillaris* is a rare and highly lethal free-living amoeba that occurs naturally in the environment and has been detected in sources such as mud, ponds, and swimming pools (4). The organism was first identified in a mandrill in 1989 (5). The diagnosis of BAE is particularly challenging. Traditionally, diagnosis has relied on the identification of trophozoites and cysts in brain tissue, the demonstration of granulomatous inflammation on histopathological examination, and direct immunofluorescence testing of brain tissue specimens or detection of *Balamuthia*-specific antibodies in serum or CSF (6). Diagnosis is further complicated by the deep location of intracranial lesions, which often makes brain biopsy technically challenging. In addition, early signs of infection may be subtle and nonspecific, potentially delaying clinical presentation and recognition of the underlying infection. Once *B. mandrillaris* invades the central nervous system, the disease can progress rapidly, resulting in severe neurological deterioration and death within a short period. Consequently, the limited diagnostic window poses a substantial challenge to timely recognition and definitive diagnosis. CSF abnormalities are likewise nonspecific, further complicating the early diagnosis of BAE. Neuroimaging can facilitate the detection of central nervous system lesions; however, the radiological findings are generally nonspecific. During the early stage of the disease, only a solitary hypodense lesion may be detectable, whereas multiple intracranial lesions typically become apparent later in the disease course (7). NGS offers the advantage of unbiased, target-independent detection of a broad range of pathogens within clinical specimens. In several reported cases, mNGS enabled the initial detection of *B. mandrillaris*, with the diagnosis subsequently confirmed by brain biopsy. These findings highlight the value of mNGS for the rapid identification of rare and unexpected pathogens, particularly in patients with severe central nervous system infections. Second, mNGS is a high-throughput sequencing approach that enables unbiased and comprehensive detection of a broad spectrum of microorganisms by sequencing DNA and RNA extracted from clinical specimens, including bacteria, viruses, fungi, and parasites. This feature makes mNGS particularly valuable for identifying rare, novel, or unexpected pathogens. In the present case, the inflammatory response was not prominent during the early stage of infection. Meanwhile, *B. mandrillaris* infection may have triggered an autoimmune response, potentially contributing to the presence of anti-NMDAR antibodies in the CSF. Consequently, the patient was initially diagnosed with anti-NMDAR encephalitis. As the disease progressed, however, the evolving clinical manifestations and laboratory findings, together with the results of mNGS, ultimately established the diagnosis of BAE. To our knowledge, BAE presenting with clinical and immunological features suggestive of anti-NMDAR encephalitis has not previously been reported. This report aims to improve clinicians’ understanding of the clinical course of *B. mandrillaris* infection and promote earlier recognition and diagnosis. Importantly, it also raises awareness that infections may trigger autoimmune responses during the early stages of disease, potentially leading to autoantibody production and clinical manifestations that mimic autoimmune encephalitis.

## Case description

**FIGURE 1:**
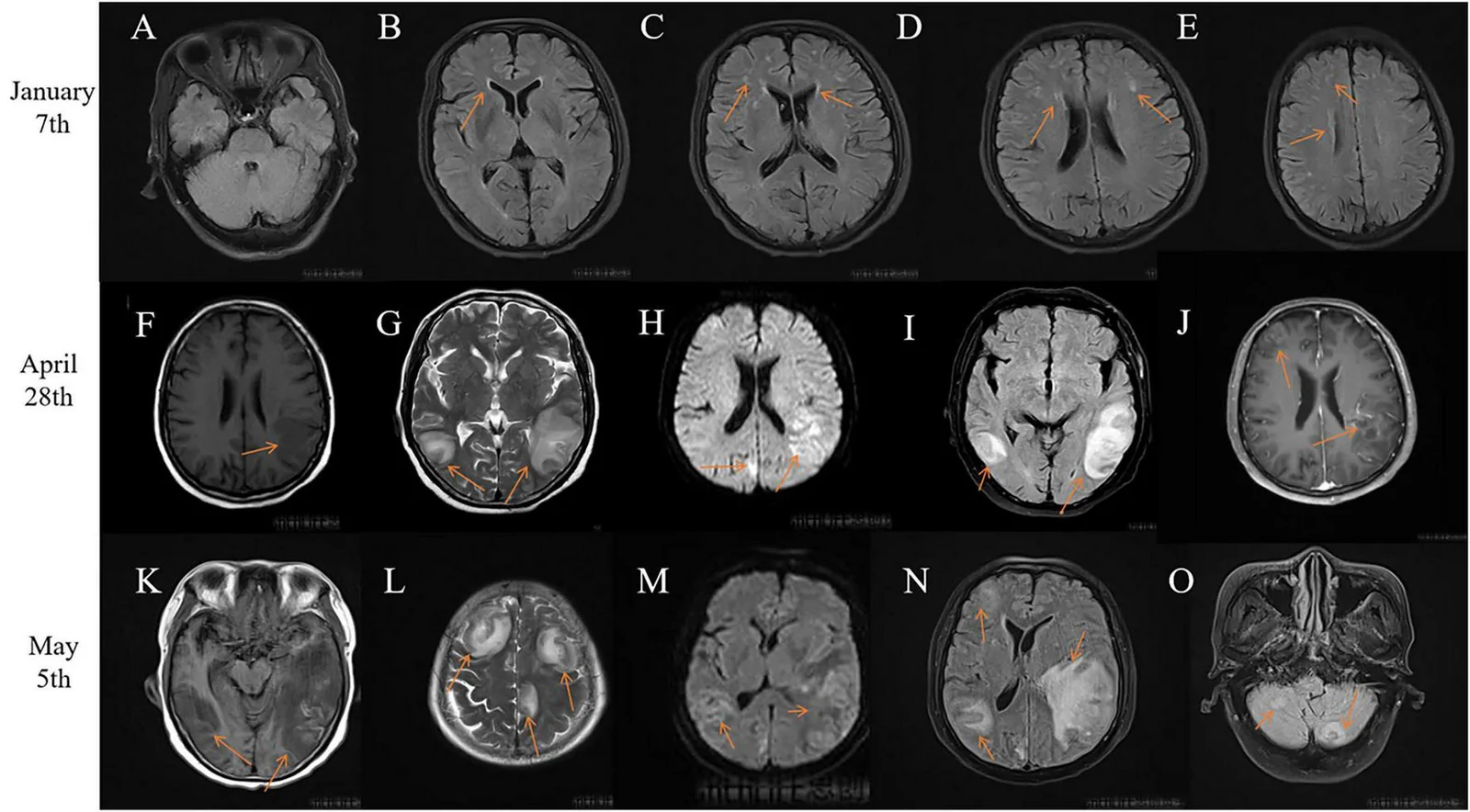
(**A–E)** Show MRI images from January 7, 2025. **(A–E)** All five FLAIR images demonstrate ischemic white matter lesions involving the bilateral frontoparietal subcortical white matter, centrum semiovale, and periventricular white matter adjacent to the lateral ventricles, April 28, 2025. **(F)** The T1-weighted image demonstrates subcortical swelling and abnormal signal intensity in the left cerebral hemisphere. **(G)** The T2-weighted images demonstrate subcortical swelling and abnormal signal intensity in both cerebral hemispheres. **(H)** Diffusion-weighted imaging demonstrates multiple patchy and mildly elongated areas of increased signal intensity involving the cortical and subcortical regions of both cerebral hemispheres, accompanied by cerebral parenchymal edema. The largest lesion is located in the left temporo-occipital region and causes compression of the temporal horn of the left lateral ventricle. **(I)** T2-weighted FLAIR images reveal multiple patchy, mildly elongated hyperintense lesions in the cortical and subcortical regions of both cerebral hemispheres. **(J)** Contrast-enhanced MRI demonstrated multiple patchy and small ring-enhancing lesions with heterogeneous enhancement in the cortical and subcortical regions of both cerebral hemispheres and in both cerebellar hemispheres, accompanied by associated parenchymal swelling, May 5, 2025 **(K–O)**. **(K)** Follow-up T1-weighted imaging demonstrates swelling of both cerebral hemispheres, with an increase in the extent of the abnormal signal-intensity lesions compared with the previous examination. **(L)** T2-weighted imaging revealed multiple areas of abnormal signal intensity in the cortices of both cerebral hemispheres, with an increase in the extent of the lesions. **(M)** DWI revealed multiple patchy and mildly elongated areas of abnormal signal intensity in the cortical and subcortical regions of both cerebral hemispheres. These lesions were mildly hyperintense on DWI and were accompanied by cerebral parenchymal swelling and increased compression of the temporal horn of the left lateral ventricle. **(N)** T2-FLAIR imaging revealed multiple patchy and mildly elongated areas of abnormal signal intensity in the subcortical regions of both cerebral hemispheres. These lesions were hyperintense on T2-FLAIR images and had increased in size. **(O)** T2-FLAIR imaging revealed new lesions in the subcortical regions of both cerebellar hemispheres.

The patient was a farmer who raised sheep for a living and presented with dizziness, headache, low-grade fever, and intermittent seizures for 5 days. Brain magnetic resonance imaging (MRI) at the referring hospital demonstrated a patchy abnormal signal in the right thalamic region, and a preliminary diagnosis of suspected intracranial infection was made. Despite 3 days of treatment with supportive measures to improve cerebral circulation and empirical anti-infective therapy, the patient continued to experience intermittent seizures and was subsequently transferred to our hospital. The patient developed intermittent seizures accompanied by loss of consciousness and was treated with intravenous ceftriaxone and acyclovir, along with adjunctive therapy to improve microcirculation and control seizures. Following admission, comprehensive laboratory and ancillary examinations were performed. Routine blood tests and serum biochemical analyses revealed no significant abnormalities, although two infection-related biomarkers were elevated. CSF analysis showed an increased white blood cell count with a predominance of mononuclear cells. Brain MRI demonstrated multiple ischemic white matter lesions (Figures 1A–E). Electroencephalography (EEG) during quiet wakefulness predominantly showed low-amplitude, fast alpha activity with irregular waveforms. Abundant low-amplitude beta activity was observed across all leads, accompanied by a small amount of low- to moderate-amplitude theta activity and occasional low-amplitude delta activity. mNGS of the CSF, incorporating both DNA and RNA sequencing, detected no pathogenic microorganisms. Neuronal autoantibody testing of the CSF revealed positivity for anti-NMDAR IgG antibodies at a titer of 1:10 (Table 1), whereas the corresponding antibodies were not detected in serum. On the basis of the clinical presentation and ancillary findings, a working diagnosis of autoimmune encephalitis was made. The patient subsequently received high-dose intravenous methylprednisolone pulse therapy at 1,000 mg/day, followed by gradual tapering and conversion to oral corticosteroid therapy. His symptoms improved substantially, his body temperature normalized, and he remained seizure-free. Following discharge, oral corticosteroids were gradually tapered, and antiseizure therapy was continued. One month after discharge, the patient returned for outpatient follow-up and reported only mild headache and fatigue, without recurrence of seizures. EEG during quiet wakefulness was predominantly characterized by low- to moderate-amplitude alpha activity with irregular waveforms. A mild increase in beta activity was observed across all leads, accompanied by a slight increase in theta activity and a small amount of low-amplitude delta activity with complex waveforms. The complete blood count (CBC) showed a mildly elevated white blood cell count of 10.94 × 10^9^/L, which may have been associated with corticosteroid therapy. Continued follow-up and monitoring were therefore recommended.

**TABLE 1:** Summary of characteristics of common central nervous system infections.

Diseases	Typical clinical course	Major clinical manifestations	Cerebrospinal fluid (CSF) findings	MRI findings	Definitive diagnosis/key diagnostic tests
Herpes simplex encephalitis (HSE)	Acute onset, developing over hours to days	Fever, behavioral and psychiatric abnormalities, memory impairment, aphasia, and focal seizures	Lymphocytic pleocytosis with elevated protein levels; red blood cells may be present, although their absence does not exclude the diagnosis	Asymmetric abnormalities involving the medial temporal lobes, insular cortex, and orbitofrontal regions; commonly associated with restricted diffusion, hemorrhage, or necrosis	CSF HSV polymerase chain reaction (PCR) assay; early testing may yield false-negative results
Autoimmune encephalitis (AE)	Subacute onset, typically evolving over days to 3 months	Psychiatric symptoms, recent memory impairment, seizures, language dysfunction, movement disorders, and autonomic instability	Mild lymphocytic pleocytosis, oligoclonal bands, or elevated IgG index; CSF findings may also be entirely normal	Limbic encephalitis may demonstrate bilateral medial temporal lobe abnormalities; MRI is often normal or nonspecific in anti-NMDA receptor encephalitis	Detection of neuronal autoantibodies in serum and/or CSF; diagnosis requires integration of clinical presentation, MRI, EEG findings, and exclusion of infectious etiologies
Central nervous system tuberculosis (CNS tuberculosis)	Subacute or chronic course	Low-grade fever, headache, altered mental status, cranial nerve palsies, hydrocephalus, and cerebral infarction; tuberculomas may present with seizures and focal neurological deficits	Elevated opening pressure, lymphocytic pleocytosis, markedly increased protein concentration, and reduced glucose levels	Basal meningeal enhancement, hydrocephalus, and basal ganglia infarctions; tuberculomas may appear as nodular or ring-enhancing lesions	CSF acid-fast bacillus staining, mycobacterial culture, and nucleic acid amplification testing; negative results do not completely exclude disease and repeated testing with larger CSF volumes is often required
Cryptococcal meningoencephalitis	Usually subacute or chronic	Headache, fever, increased intracranial pressure, and altered consciousness; invasive fungal infections such as aspergillosis may cause acute focal neurological deficits or cerebral infarction	Markedly elevated opening pressure and decreased glucose levels are typical in cryptococcal infection; CSF cell counts may be low in severely immunocompromised patients	Leptomeningeal enhancement, cryptococcomas, or enlarged perivascular spaces may be observed; aspergillosis may present with multiple infarcts, hemorrhage, or abscess formation	Cryptococcal antigen testing, fungal culture, fungal PCR, or metagenomic next-generation sequencing (mNGS)
Toxoplasma encephalitis	Commonly occurs in patients with advanced HIV infection, organ transplantation, or severe immunosuppression	Fever, headache, altered mental status, seizures, and focal neurological deficits	Mild elevation of white blood cell count and protein concentration; glucose levels are usually normal	Multiple ring-enhancing lesions, commonly involving the basal ganglia, thalamus, and gray-white matter junction, with prominent surrounding edema	Serum Toxoplasma IgG supports previous exposure; CSF PCR positivity is highly suggestive but has limited sensitivity; brain biopsy may be required for definitive diagnosis
Primary central nervous system lymphoma (PCNSL)	Usually subacute onset	Cognitive impairment, psychiatric changes, focal neurological deficits, and increased intracranial pressure; fever is uncommon	Mild-to-moderate elevation of CSF white blood cells and protein; glucose is usually normal; malignant cells may occasionally be detected	In immunocompetent patients, lesions are commonly located in deep brain structures, periventricular regions, or the corpus callosum, showing intense homogeneous enhancement and restricted diffusion; immunocompromised patients may present with atypical ring-enhancing lesions	Stereotactic brain biopsy remains the cornerstone for definitive diagnosis
Bacterial brain abscess	Acute to subacute course	Fever, headache, focal neurological deficits, seizures, and increased intracranial pressure; the classic triad is not always present	CSF examination has limited diagnostic value; CSF cell counts may be normal when the abscess is well localized	Smooth-walled ring-enhancing lesions with marked central diffusion restriction within the abscess cavity on diffusion-weighted imaging (DWI), which is an important diagnostic clue	Surgical aspiration or drainage specimens for Gram staining, bacterial culture, anaerobic culture, and molecular testing
Acanthamoeba encephalitis (granulomatous amoebic encephalitis)	Weeks to months	Headache, low-grade fever, behavioral changes, seizures, ataxia, cranial nerve involvement, and focal neurological deficits	Lymphocytic or mixed-cell pleocytosis with elevated protein levels; glucose may be normal or decreased; findings are often nonspecific	Multifocal space-occupying lesions with ring or nodular enhancement, sometimes accompanied by hemorrhage, necrosis, and extensive edema	Histopathological examination, immunohistochemistry, and PCR of brain tissue, skin lesions, or other infected tissues; CSF PCR may provide supportive evidence
*Naegleria fowleri* primary amebic meningoencephalitis (PAM)	Fulminant course; typically develops within several days after exposure to warm freshwater entering the nasal cavity; history of nasal irrigation exposure should also be considered	Severe headache, high fever, vomiting, neck stiffness, followed by rapidly progressive altered consciousness, seizures, cerebral edema, and coma	Similar to bacterial meningitis: elevated opening pressure, marked neutrophilic pleocytosis, increased protein levels, and decreased glucose; motile trophozoites may be detected in fresh CSF samples	Early MRI findings may be nonspecific; later findings include diffuse cerebral edema, meningeal enhancement, or hemorrhagic changes	CSF wet mount examination, staining, PCR testing, or tissue-based molecular detection; disease progression is typically rapid over several days
*Balamuthia* amebic encephalitis (BAE)	Subacute to chronic course; may occur in immunocompetent individuals and may be associated with soil exposure or chronic skin lesions	Headache, fever, behavioral changes, seizures, hemiparesis, ataxia, and cranial nerve deficits	Elevated protein concentration and lymphocytic pleocytosis; glucose levels are usually normal	Multiple hemorrhagic, necrotic, or ring-enhancing lesions that may mimic tumors, vasculitis, or brain abscesses	Histopathology, immunohistochemistry, and PCR of skin or brain tissue; definitive diagnosis usually relies on tissue specimens

Three months after the initial presentation, the patient developed recurrent headache accompanied by fever for 4 days, with a maximum body temperature of 38.3 °C. He also exhibited slurred speech and slowed responsiveness. On readmission, neurological examination revealed impairment in higher cortical functions on bedside assessment, including memory, calculation, judgment, and orientation. The pupils were equal, round, and approximately 3 mm in diameter bilaterally, with brisk responses to light. Extraocular movements were intact, and no nystagmus was observed. Meningeal signs were absent. Muscle strength in the right extremities was graded as 4/5. Superficial and deep sensation and coordination could not be reliably assessed because of limited patient cooperation. No pathological reflexes were elicited. MRI demonstrated multifocal cortical and subcortical swelling with abnormal signal intensities involving both cerebral hemispheres (Figures 1F–J). A retrospective review of the patient’s exposure history revealed that, shortly before his initial presentation, several sheep from his flock had become ill and exhibited abnormal aggressive behavior, including marked agitation and biting. The affected animals subsequently died without receiving formal veterinary treatment, and the patient personally handled and buried the carcasses. In view of the patient’s clinical history and current presentation, a working diagnosis of intracranial infection was made, and empirical anti-infective therapy with intravenous acyclovir (1.5 g/day) and ceftriaxone (2 g/day) was initiated. Intravenous methylprednisolone (IVMP) was also administered because of the previously suspected autoimmune encephalitis.

**FIGURE 2:**
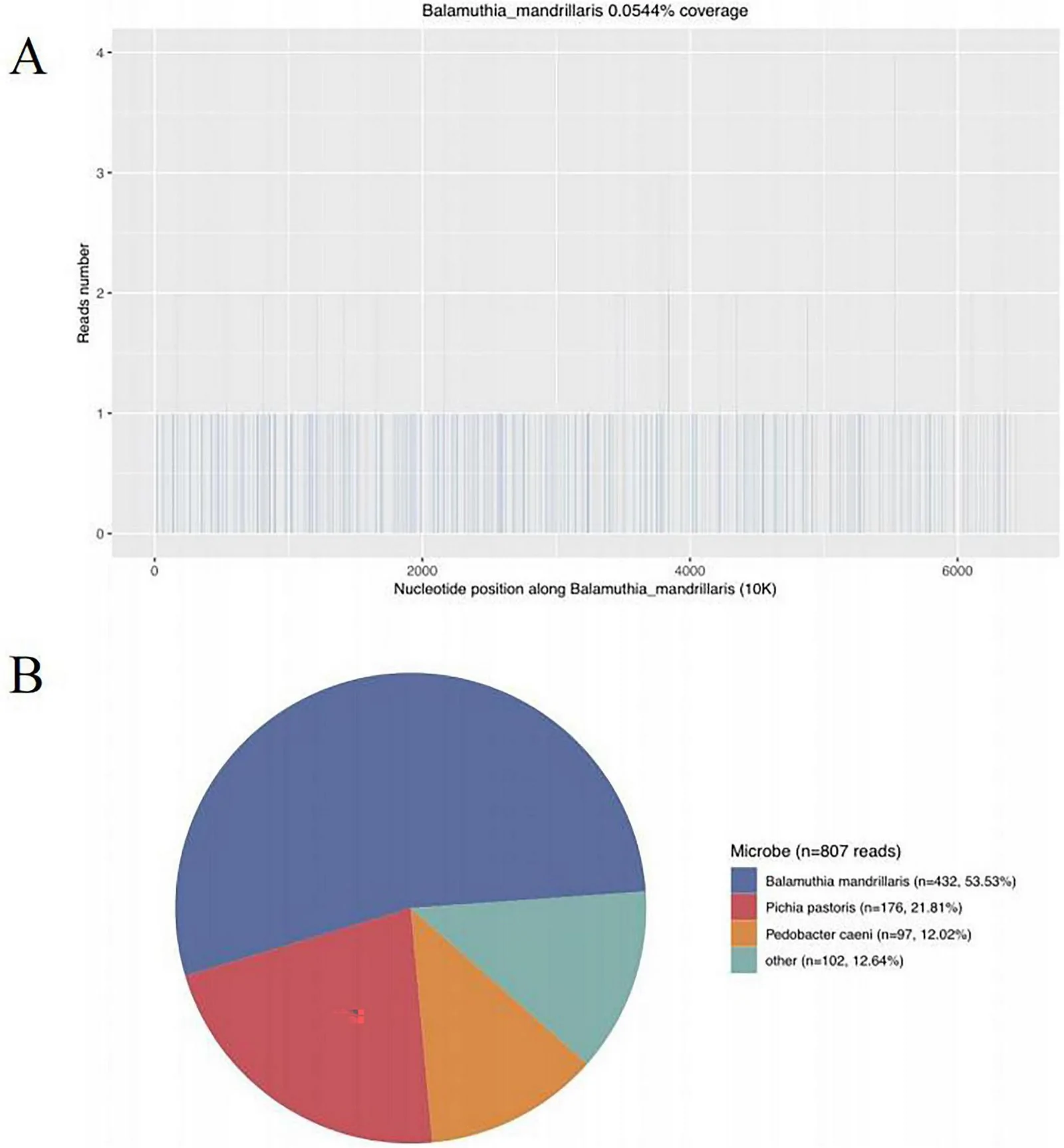
**(A)** Metagenomic next-generation sequencing identified 432 specific reads of *Balamuthia mandrillaris*, and the figure shows the genomic location distribution of the detected sequences. **(B)** Read composition of microbes in the CSF sample.

Cerebrospinal fluid examination revealed a clear, colorless appearance and an opening pressure of 170 mmH_2_O. CSF biochemical analysis showed a glucose level of 2.51 mmol/L, a chloride level of 117.30 mmol/L, and an elevated protein concentration of 1.02 g/L. The CSF white blood cell count was increased to 34 × 10^6^/L, with mononuclear cells accounting for 99% of the total leukocyte count. On May 1, mNGS of the CSF identified *B. mandrillaris*, with 432 unique reads specifically mapping to *B. mandrillaris* and a relative abundance of 95%. Based on the patient’s clinical manifestations, laboratory findings, brain MRI findings, and the mNGS results, a definitive diagnosis of BAE was established. The genomic coverage of *B. mandrillaris* identified by mNGS is shown in Figure 2. Following the diagnosis, combination antimicrobial therapy was initiated immediately, consisting of ornidazole (0.5 g/day), voriconazole (0.36 g/day), azithromycin (0.5 g/day), trimethoprim–sulfamethoxazole (TMP–SMX; 1.6 g), and flucytosine (1.5 g). Despite this multidrug regimen, the patient’s clinical condition continued to deteriorate progressively.

After 1 week of combination antimicrobial therapy, the patient deteriorated into a comatose state. Repeat brain MRI demonstrated multifocal cortical and subcortical swelling with abnormal signal intensities involving both cerebral hemispheres and both cerebellar hemispheres. Compared with the previous MRI findings, the pre-existing lesions had increased in size, and multiple new lesions had developed in the bilateral cerebellar hemispheres (Figures 1K–O). Despite the progressive deterioration, the patient’s family declined further treatment and requested discharge. The patient died 1 week after discharge.

## Discussion

*Balamuthia mandrillaris* is one of the free-living amoebae known to cause severe infections of the central nervous system. Infection may initially manifest as cutaneous lesions; however, once the organism invades the brain, it can cause severe and often fatal BAE. *B. mandrillaris* is widely distributed in the environment, particularly in soil and water. A search of the PubMed database using the term “*Balamuthia* amoebic encephalitis” identified 283 reported cases. However, we found no previously reported cases in which *B. mandrillaris* infection was associated with autoimmune manifestations resembling autoimmune encephalitis. To our knowledge, the present case is the first reported case of BAE associated with anti-NMDAR antibody positivity and clinical features mimicking anti-NMDAR encephalitis, highlighting the exceptional rarity of this presentation.

**FIGURE 3:**
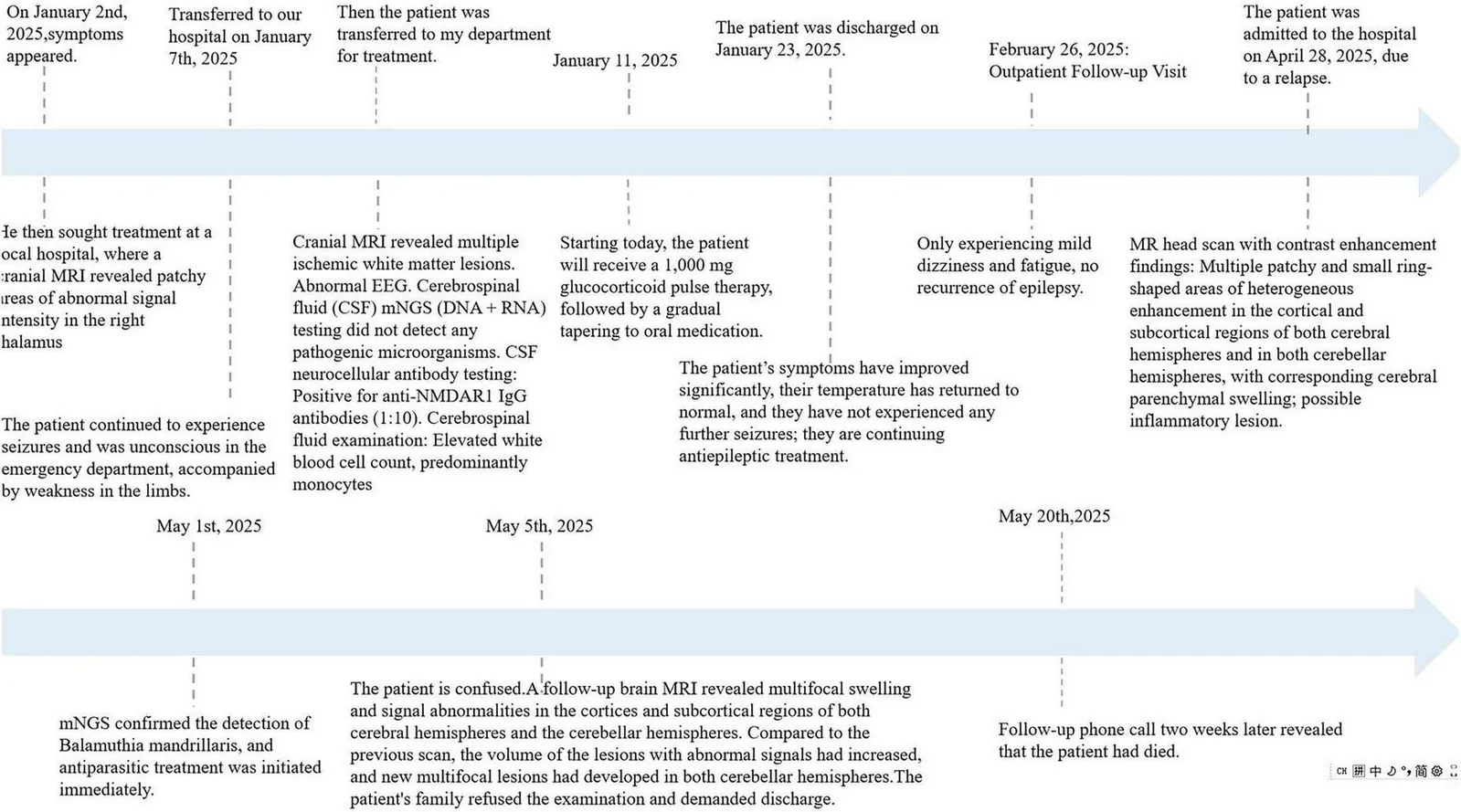
Changes in the disease over time and the diagnosis and treatment process.

A chronological overview of the patient’s disease course, including disease progression, diagnostic evaluation, therapeutic interventions, and clinical outcomes, is presented in Figure 3. A review of the patient’s medical history revealed no preceding symptoms of upper respiratory tract infection or fever and no history of immune-mediated disease. However, shortly before the onset of illness, the patient had handled sheep exhibiting abnormal behavioral or neurological manifestations and had buried the carcasses directly in soil without incineration or other appropriate disposal measures. This exposure history suggests a substantial risk of environmental exposure to the parasite. We therefore hypothesize that cutaneous and/or respiratory contact with contaminated soil may have facilitated the entry of amoebic cysts into the body, followed by invasion of the subcutaneous tissues and/or lungs, where they may have persisted in a latent state.

The patient subsequently developed prominent neurological manifestations, including seizures, memory impairment, altered consciousness, and abnormalities in body temperature regulation. In conjunction with CSF positivity for anti-NMDAR antibodies, these findings strongly supported a working diagnosis of anti-NMDAR encephalitis at the initial presentation. Moreover, the initial neuroimaging findings and the substantial clinical response to corticosteroid therapy were consistent with an immune-mediated neurological process. Considering the subsequent disease course, we speculate that *B. mandrillaris* infection may already have been present at this stage but had not yet resulted in overt central nervous system involvement. Alternatively, the amoebic burden within the CNS may have been too low to be detected by the initial CSF mNGS assay. This possibility raises the hypothesis that an infection-associated autoimmune response may have preceded clinically apparent BAE.

The initially negative CSF mNGS result may be explained by the following factors. First, the parasite may have remained latent in peripheral tissues without invading the CNS, thereby resulting in a negative CSF mNGS result. This is considered the most likely explanation in this patient. Second, the sequencing depth may have been insufficient. At the time of lumbar puncture, the patient had an impaired level of consciousness and was poorly cooperative, which made the procedure technically challenging. The collected CSF was slightly blood-tinged, and subsequent laboratory examination revealed the presence of red blood cells together with an elevated white blood cell count. Such cellular contamination may have increased the proportion of host-derived genomic DNA in the sample, thereby reducing the relative abundance of pathogen-derived nucleic acids and consequently decreasing the sensitivity of mNGS for pathogen detection. In a large clinical cohort, among cases in which pathogens were missed by mNGS but detected by conventional direct testing of CSF, approximately 38.5% of the false-negative mNGS results were associated with a high background of human-derived DNA, which reduced the sensitivity of pathogen detection (8). Consequently, although the total sequencing depth may appear adequate, the effective sequencing depth available for microbial reads may remain insufficient. Third, suboptimal DNA extraction efficiency may also have contributed to the initial false-negative mNGS result. If the lysis procedure was not sufficiently effective for *B. mandrillaris*, the cyst wall may not have been adequately disrupted, resulting in incomplete release of pathogen DNA and, consequently, an insufficient amount of *B. mandrillaris*-derived DNA in the final sequencing library. To date, studies directly quantifying the effects of different nucleic acid extraction methods on the sensitivity of mNGS for detecting *B. mandrillaris* are lacking. Therefore, this explanation remains a biologically and technically plausible hypothesis based on the principles and methodological limitations of mNGS rather than a mechanism directly demonstrated in the present case. Fourth, a low pathogen burden in the CSF may also contribute to false-negative mNGS results. In BAE, the pathogen burden may be substantially higher in the brain parenchyma than in the CSF, resulting in only a very small number of pathogen-derived reads in CSF samples. In one reported case, retrospective analysis of the raw CSF mNGS data identified only two reads corresponding to *B. mandrillaris*, whereas examination of brain tissue yielded a definitive positive result. This case illustrates how a low pathogen burden in the CSF, combined with predefined reporting thresholds, may result in a clinically reported negative mNGS result despite the presence of detectable pathogen-derived sequences in the raw sequencing data (9).

We further speculate that uneven pathogen distribution may also contribute to false-negative results. In the same patient, the number of pathogen-derived sequences detected in CSF may vary considerably depending on the timing and anatomical site of sampling. It is also possible that only one or two amoebic cells, or a very small amount of pathogen-derived DNA, were present in the original specimen and were progressively lost during sample processing, nucleic acid extraction, library preparation, and sequencing. Fifth, a mismatch between the sampling site and the actual site of infection may reduce the diagnostic yield of CSF mNGS. If CSF is collected before overt central nervous system involvement has developed, the result may remain negative. Similarly, when the specimen type does not adequately represent the true site of infection, pathogen detection may fail. Large clinical studies of mNGS in central nervous system infections have shown that some pathogens can be detected only in brain tissue, abscess fluid, or other non-CSF specimens. *B. mandrillaris* has also been reported among pathogens that may be missed by CSF mNGS but subsequently identified in non-CSF specimens. These observations highlight the importance of selecting specimens that most closely correspond to the suspected anatomical site of infection when interpreting negative mNGS findings. Sixth, the timing of lumbar puncture may have been suboptimal. If CSF is collected too early in the course of infection, the pathogen may have only recently invaded the brain parenchyma, before substantial tissue injury or release of pathogen-derived DNA into the CSF has occurred. Consequently, the pathogen signal in the CSF may remain extremely low or even undetectable. Moreover, the release of pathogen-derived nucleic acids into the CSF may be intermittent or fluctuate over time. A single lumbar puncture performed during a period of low pathogen shedding may therefore yield a negative result, whereas repeat sampling several days later may detect sufficient pathogen-derived sequences for identification. A recent report described a case in which the CSF specimen was collected 7 days before plasma mNGS subsequently yielded a positive result. The authors suggested that this temporal discrepancy may have contributed to the negative CSF finding and that simultaneous collection of CSF and plasma might have produced different results. This observation further highlights the potential influence of sampling timing on the diagnostic yield of mNGS. Seventh, bioinformatic filtering may contribute to false-negative mNGS results. Because both free-living amoebae and humans are eukaryotic organisms, certain highly conserved sequences may share a degree of similarity. During bioinformatic filtering, some genuine amoeba-derived reads may therefore be misclassified as human-derived or low-complexity sequences and subsequently removed. In addition, reads originating from repetitive regions, low-complexity regions, or highly conserved rRNA sequences within the amoebic genome may be directly filtered out. Although such filtering procedures are important for reducing false-positive signals, they may also inadvertently eliminate the few informative pathogen-derived reads present in samples with a very low pathogen burden, thereby increasing the risk of false-negative mNGS results. Eighth, an overly stringent reporting threshold may contribute to false-negative mNGS results. In a previously reported case, two sequencing reads corresponding to *B. mandrillaris* were identified in the raw CSF mNGS data; however, because the laboratory had adopted a reporting threshold of 50 reads, the initial mNGS result was reported as negative. Reporting criteria and cutoff values can vary considerably across laboratories, and such differences may contribute to discordance between low-level pathogen signals in the raw sequencing data and the final clinical report (9). Further evidence supporting this possibility comes from a study evaluating 7 years of clinical mNGS implementation. In that study, 35 pathogen signals were detected below the conventional reporting threshold, of which 32 were ultimately considered to represent true infections. In addition, three false-negative mNGS cases were subsequently found to contain unreported pathogen signals below the predefined reporting cutoff. These findings suggest that stringent reporting thresholds may occasionally result in clinically relevant low-abundance pathogen signals being overlooked, particularly when the pathogen burden is low (8). Ninth, contamination-control procedures may indirectly contribute to false-negative mNGS results. When only a very small number of *Balamuthia mandrillaris*-derived reads are present in a patient sample, while several reads corresponding to the same organism are also detected in the negative control because of low-level cross-contamination, certain bioinformatic filtering algorithms may consider the difference between the clinical specimen and the background control insufficient to support a true-positive result. Consequently, a genuine low-abundance pathogen signal may be classified as background contamination and excluded from the final clinical report. Tenth, the diagnostic sensitivity and specificity of mNGS for *B. mandrillaris* remain insufficiently characterized. To date, no sufficiently large-scale studies have established reliable estimates of the sensitivity and specificity of CSF mNGS for the diagnosis of BAE. Therefore, a negative CSF mNGS result should be interpreted cautiously and should not, in isolation, be considered sufficient to exclude *B. mandrillaris* infection, particularly when the clinical course, neuroimaging findings, or epidemiological exposure history remains suggestive of this diagnosis. The currently available evidence is derived predominantly from case reports, small case series, and retrospective reviews of published cases, all of which are inherently susceptible to substantial publication and selection bias. Previous studies have also emphasized that the sensitivity of CSF mNGS for detecting *B. mandrillaris* remains uncertain (9). In light of the potential limitations discussed above, we therefore propose that, for rare pathogens such as *B. mandrillaris*, a negative CSF mNGS result should be interpreted as indicating that the pathogen signal in the tested specimen did not reach the analytical detection or reporting threshold, rather than as definitive evidence that infection has been excluded.

The detection of anti-NMDAR antibodies in the CSF during the early stage of the patient’s illness suggests a mechanism more complex than simple coexistence of infection and autoimmune encephalitis. Viral infections are recognized as potential triggers of anti-NMDAR encephalitis, raising the possibility that infection with a free-living amoeba such as *B. mandrillaris* could induce a similar infection-associated autoimmune response. Based on currently proposed mechanisms of infection-induced autoimmunity, we speculate that several processes may have contributed to the development of anti-NMDAR antibodies in this patient. First, *B. mandrillaris* infection may have promoted peripheral activation of autoreactive B cells. The normal human B-cell repertoire can contain a small population of autoreactive B-cell clones capable of weakly recognizing self-antigens. Under physiological conditions, these cells are generally maintained in a state of immunological tolerance; however, infection-associated immune activation may potentially provide the inflammatory and costimulatory signals necessary for their activation and expansion. Under physiological conditions, these autoreactive B cells are restrained by multiple mechanisms of immune tolerance, including regulatory T-cell-mediated suppression and the absence of sufficient costimulatory signals, and therefore do not necessarily give rise to autoimmune disease. During parasitic infection, however, pathogen-associated molecules, including parasitic DNA, RNA, and glycolipid components, together with an inflammatory milieu characterized by cytokines such as interleukin-6 (IL-6) and interferon-γ (IFN-γ), B-cell survival and activation factors such as B-cell activating factor (BAFF) and a proliferation-inducing ligand (APRIL), and persistent exposure to parasitic antigens, may provide multiple and sustained immunostimulatory signals. These signals may not only activate parasite-specific B cells but also lower the activation threshold of previously quiescent autoreactive B-cell clones, promoting their activation, expansion, and differentiation into antibody-producing cells and thereby potentially facilitating the production of multiple autoantibodies (10, 11). Previous studies have shown that, during *Plasmodium falciparum* infection, infected erythrocytes can directly interact with B cells and promote polyclonal immunoglobulin production. Both clinical and experimental studies have further demonstrated that malaria is associated with polyclonal B-cell activation, hypergammaglobulinemia, and the production of a broad range of autoantibodies. By analogy, we speculate that *B. mandrillaris*, as a pathogenic free-living amoeba, may induce B-cell activation through partially overlapping immunological pathways, thereby promoting polyclonal antibody responses and potentially facilitating the activation of autoreactive B-cell clones. Although this mechanism has not been directly demonstrated in *B. mandrillaris* infection, we consider infection-induced polyclonal B-cell activation to be one of the more biologically plausible explanations for the emergence of autoantibodies in the present case. Second, molecular mimicry between *B. mandrillaris* antigens and the GluN1 subunit of the NMDAR may represent another potential mechanism. In theory, if parasite-derived antigens share sufficient similarity with host proteins in amino acid sequence, three-dimensional conformation, surface charge distribution, or other structural features, B-cell antibodies or T-cell responses initially directed against the pathogen could cross-react with host antigens. In this context, immune responses generated against *B. mandrillaris* might potentially cross-recognize epitopes within the GluN1 subunit of the NMDAR (12, 13). In anti-NMDAR encephalitis, the pathogenic autoantibodies predominantly recognize conformational epitopes located within the extracellular amino-terminal domain of GluN1. We therefore consider molecular mimicry between *B. mandrillaris* antigens and GluN1 to be a biologically plausible hypothesis that warrants further investigation through structural, immunological, and functional studies. Third, blood–brain barrier (BBB) dysfunction may occur at a level that is not detectable by conventional MRI. Routine MRI sequences and contrast-enhanced MRI primarily reveal macroscopic abnormalities such as edema, necrosis, extensive inflammation, structural tissue injury, or demyelination, whereas subtle functional alterations in BBB integrity may remain radiologically occult. Such changes may involve redistribution or disruption of tight-junction proteins, increased endothelial permeability, enhanced leukocyte adhesion and transendothelial migration, and localized, transient, or low-level passage of circulating antibodies into the CNS. Evidence from malaria provides a potentially relevant mechanistic analogy. During *Plasmodium* infection, inflammatory mediators such as tumor necrosis factor-α (TNF-α), interferon-γ (IFN-γ), and interleukin-1β (IL-1β) may activate cerebral microvascular endothelial cells, promote reorganization of endothelial tight junctions, increase vascular permeability, and facilitate leukocyte recruitment (14, 15). However, the precise molecular mechanisms responsible for BBB disruption during malaria remain incompletely understood. By analogy, we speculate that infection-associated inflammatory responses in *B. mandrillaris* infection might induce subtle BBB dysfunction sufficient to permit limited entry of B cells, plasmablasts, or circulating antibodies into the CNS, even in the absence of overt abnormalities attributable to BBB disruption on conventional MRI. Importantly, previous studies of anti-NMDAR encephalitis have demonstrated CD138-positive plasma cells within brain tissue and perivascular spaces. Clonally expanded plasma cells exhibiting somatic hypermutation have also been identified in the cerebrospinal fluid, supporting the presence of antigen-driven B-cell maturation and local antibody production within the CNS. Taken together, these observations provide a biologically plausible framework for the findings in our patient: anti-NMDAR antibodies were clearly detectable in the CSF despite being undetectable in serum, while no *B. mandrillaris*-derived signal was identified in the CSF during the early stage of the disease. We therefore speculate that subtle BBB dysfunction, together with compartmentalized intrathecal B-cell activation and antibody production, may have contributed to this apparently discordant immunological profile. Fourth, fever, inflammation, and seizure activity may further compromise BBB integrity. Clinical studies have shown that status epilepticus can be associated with alterations in BBB permeability (16). In patients with anti-NMDAR encephalitis, increased levels of markers associated with BBB disruption, including matrix metalloproteinase-9 (MMP-9), have also been reported, suggesting that BBB dysfunction may participate in the pathophysiology of the disease (17). On this basis, we propose a possible sequential mechanism in the present case. *B. mandrillaris* infection may initially have triggered fever, systemic or neuroinflammation, and seizures. Seizure-associated BBB dysfunction could then have increased barrier permeability and promoted the exposure or release of neuronal antigens. Under these conditions, GluN1-reactive B cells or plasmablasts might have gained greater access to the central nervous system and undergone further antigen-driven stimulation, thereby amplifying the intrathecal anti-GluN1 antibody response. Although this proposed sequence remains speculative and cannot be established from a single case, it provides a biologically plausible explanation for the progressive development of the autoimmune response observed in this patient.

The clinical manifestations of BAE are largely nonspecific. Therefore, we summarized the characteristic features of several common clinical disorders that may present with symptoms similar to those observed in our patient (Table 1). At the initial onset of the disease, brain MRI revealed only a few ischemic white matter lesions in the periventricular regions, with no other significant abnormalities. As the disease progressed, however, repeat brain MRI during the second hospitalization demonstrated multiple areas of swelling involving the cortical and subcortical regions of both cerebral hemispheres. An infectious process was subsequently suspected, prompting contrast-enhanced MRI, which revealed nodular and small ring-like lesions with heterogeneous enhancement, accompanied by swelling of the corresponding brain regions. We speculate that corticosteroid therapy may have compromised the patient’s immune defenses, thereby facilitating the dissemination of previously latent amoebae from peripheral tissues to the central nervous system and ultimately contributing to the marked interval changes observed on neuroimaging. Brain MRI findings in BAE do not show a strictly defined anatomical predilection. Reported imaging features commonly include nodular or ring-enhancing lesions, which were also observed in our patient (18). Diffusion restriction was present within the corresponding areas of swelling, and the larger lesion exerted a mass effect with compression of the temporal horn of the left lateral ventricle. On the third brain MRI examination, the previously identified lesions had further increased in size, with more pronounced surrounding swelling compared with the preceding study. Given the unusual clinical course of this patient, we further considered the potential relationship between autoimmune encephalitis and BAE. The most characteristic MRI abnormalities associated with autoimmune encephalitis, particularly anti-NMDAR encephalitis, include T2-weighted and fluid-attenuated inversion recovery (FLAIR) hyperintensity and swelling involving the hippocampus, amygdala, and medial temporal lobes. These imaging features differ from the progressive multifocal cortical and subcortical lesions with nodular or ring-like enhancement observed during the later stage of disease in our patient. A notable feature of anti-NMDAR encephalitis is that severe clinical manifestations may occur despite a normal or only minimally abnormal conventional brain MRI. Previous reviews have shown that MRI findings in anti-NMDAR encephalitis are most commonly normal or nonspecific, with only a minority of patients demonstrating T2-weighted or FLAIR abnormalities involving the medial temporal lobes or other brain regions (19, 20). At the initial presentation, our patient likewise showed no characteristic MRI lesions suggestive of encephalitis, which was consistent with this recognized imaging pattern. In contrast, the MRI features that subsequently developed in BAE were markedly different. Considering the patient’s clinical manifestations, relevant exposure history, rate of disease progression, serial neuroimaging findings, and microbiological results, several more common causes of encephalitis became less likely. Ultimately, the most compelling etiological evidence was provided by mNGS, which identified *B. mandrillaris* in the cerebrospinal fluid and thereby established the causative organism. However, a major limitation of the present case is that brain tissue biopsy and histopathological examination were not performed, precluding pathological confirmation of BAE.

A PubMed search identified 124 reported patients with BAE. After screening, we summarized the clinical characteristics of 90 patients, including mortality, diagnostic methods, initial clinical manifestations, treatment regimens, and clinical outcomes (Table 2). The overall mortality rate was as high as 84%. All patients exhibited evident central nervous system and/or cutaneous involvement. In most cases, the diagnosis was established by detecting *B. mandrillaris* in brain or skin tissue (68 cases), whereas 19 cases were diagnosed using next-generation sequencing (NGS). The reported cases encompassed all age groups, ranging from infants and young children to elderly patients. Notably, the initial manifestations in our patient were similar to those described in previous reports and were largely nonspecific, predominantly including headache, seizures, and fever. As the disease progressed, neuroimaging revealed space-occupying effects accompanied by irregular contrast enhancement. Among the 90 patients reviewed, those with delayed diagnoses were frequently treated empirically with antituberculosis agents and broad-spectrum antimicrobial therapy, generally with poor clinical responses. In contrast, all patients who achieved favorable outcomes received combination therapy, and the therapeutic regimens were relatively similar across these successfully treated cases. This may be attributable to the lack of a specific effective treatment for *B. mandrillaris* infection, with multidrug combination therapy potentially providing synergistic antimicrobial effects and enhancing therapeutic efficacy within the central nervous system.

**TABLE 2:** A summary of symptoms, imaging findings, diagnostic methods, treatment, and outcomes in 90 patients with *Balamuthia* amebic encephalitis.

Case	Presenting symptoms	Initial imaging findings	Diagnostic methods	Treatment	Clinical outcome
1 (26)	Right frontal area is sore, accompanied by dizziness, nausea and vomiting. There is a history of skin injury in the past.	Multiple intracranial lesions demonstrated marked, heterogeneous ring enhancement, with extensive surrounding vasogenic edema.	mNGS	Azithromycin, flucytosine, fluconazole, trimethoprim–sulfamethoxazole	Not specified
2 (27)	Fever, accompanied by weakness in the lower limbs and impaired consciousness	Patchy hyperintensity in both cerebral cortices; patchy hyperintensity is seen on the left side of the corpus callosum on diffusion-weighted imaging (DWI).	mNGS	Mechanical ventilation, acyclovir, ceftriaxone, azithromycin, methylprednisolone sodium succinate, and mannitol	Deceased
3 (28)	Moderate swelling pain in the right frontoparietal region	There is a space-occupying lesion in the right frontal lobe.	mNGS	Azithromycin, fluconazole, fluorouracil, trimethoprim–sulfamethoxazole	Deceased
4 (29)	Weakness on the left side of the body and slurred speech	There is a lesion with enhanced margins in the right parietal lobe, surrounded by vasogenic edema.	Tissue biopsy	Miltefosine, fluconazole, fluorouracil, azithromycin, and sulfadiazine	Deceased
5 (30)	Dizziness and unsteadiness while walking	Abnormal signals in the left temporal lobe	mNGS	Moxifloxacin, minocycline, and ganciclovir	Deceased
6 (2)	Symptoms of intermittent bilateral temporal headaches, accompanied by projectile vomiting	The mass has indistinct borders and shows heterogeneous enhancement.	Direct sequencing of DNA extracted from brain tissue and cerebrospinal fluid	Pentamidine, sulfasalazine, fluconazole, clarithromycin, and amphotericin B	Deceased
7 (31)	Progressive confusion	There are areas of low density in both frontal lobes, with surrounding enhancement.	Polymerase chain reaction test	Surgical treatment	Deceased
8 (25)	Numbness and weakness in the right upper limb, speech difficulties, and headache	There is a high-signal lesion in the left parietal lobe. It shows marginal enhancement and is surrounded by marked edema.	Metagenomic capture sequencing	Fluconazole, ornidazole, azithromycin, albendazole, and dexamethasone	Survived
9 (32)	Headaches, vision problems, and seizures	There is a mass in the left frontal lobe and a mass in the right temporal lobe. There is vascular edema surrounding both of these areas.	Pathological testing	Fluconazole, amantadine, fluorouracil, and clarithromycin	Survived
10 (33)	Ataxia, dizziness, nausea, and vomiting	Brain and brainstem mass, accompanied by perivascular edema	Pathological testing	Pentamidine, TMP-SMX, flucytosine, fluconazole, miltefosine, and azithromycin	Deceased
11 (34)	Fever accompanied by headache, behavioral abnormalities, and impaired consciousness	There are mass-like abnormal signals in the right frontal, parietal, and temporal lobes.	mNGS	Voriconazole, meropenem, acyclovir, vancomycin, and surgical treatment	Deceased
12 (35)	Fever, accompanied by abdominal pain, nausea, and vomiting.	Abnormal signals in the left parietal lobe	mNGS	Antiinfective and symptomatic treatment to reduce intracranial pressure	Deceased
13 (36)	Altered level of consciousness, slurred speech, and facial muscle weakness	Abnormal signals are present around the left thalamus and the thalamic cystic structures.	Real-time PCR DNA analysis	Trimethoprim–sulfamethoxazole, trimethoprim, and voriconazole	Deceased
14 (37)	Confusion	There are brain lesions in the right frontal lobe, the left basal ganglia, and the lateral aspect of the left cerebellar hemisphere.	Immunohistochemical and real-time PCR tests	Pentamidine, sulfadiazine, azithromycin, fluconazole, flucytosine, and miltefosine	Deceased
15 (38)	Symptoms of amnesia	Contrast enhancement is observed in the right thalamus and right frontal lobe, accompanied by perilesional edema.	Pathological testing	Steroid medications	Deceased
16 (39)	A subcutaneous lump on the right thigh and seizures	Right temporal lobe mass, right occipital lobe mass	Pathological testing	Broad-spectrum antibiotic therapy, cyclophosphamide	Deceased
17 (18)	Dizziness, nausea, and vomiting	Abnormal signals are present in the right temporal lobe.	mNGS	Amphotericin B, fluorouracil, fluconazole, trimethoprim–sulfamethoxazole, trimethoprim, clarithromycin, pentamidine, and intravenous immunoglobulin	Deceased
18 (40)	Vomiting, dizziness, seizures, and ataxia	A large, ill-defined, infiltrative, high-signal lesion is present in the right temporo-parietal lobe.	Conventional PCR	Rifampin, metronidazole, and miltefosine	Deceased
19 (41)	Headache and fever	There is marked thickening of the meninges, and lesions are also present in the vermis of the cerebellum.	mNGS	Ceftriaxone, fluconazole, and amphotericin B, trimethoprim–sulfamethoxazole, and metronidazole	Deceased
20 (42)	Fever and altered level of consciousness	Multiple lesions in the frontal and occipital lobes	mNGS	Amphotericin B, fluconazole, sulfamethoxazole, azithromycin, and fluorouracil	Deceased
21 (1)	Dizziness	Abnormal signals were detected in multiple regions of the brain	mNGS	Amphotericin B, cytosine, fluconazole, trimethoprim–sulfamethoxazole, trimethoprim, clarithromycin, pentamidine, and intravenous immunoglobulin	Deceased
22 (24)	Headache, nausea, and vomiting	Abnormal enhancement, ring-shaped heterogeneous enhancement, and a distinct band of edema	mNGS	Azithromycin, metronidazole, fluorouracil, fluconazole, and trimethoprim–sulfamethoxazole	Deceased
23 (43)	Seizures and headaches	Abnormally enhanced signals in the left temporal and occipital lobes	PCR testing	Pentamidine, sulfadiazine, azithromycin, fluconazole, flucytosine, and miltefosine	Survived
24 (44)	Headache	There is a poorly defined lesion with low signal intensity in the cortex and white matter of the left parietal lobe.	On real-time polymerase chain reaction	Medications for amoebic infections and dexamethasone	Deceased
25 (45)	Fever and impaired consciousness	There are ill-defined abnormal areas in both frontal lobes.	MetaCAP	Albendazole and fluconazole	Deceased
26 (46)	Fatigue, night sweats, coughing, and confusion	There are scattered areas of signal enhancement in the cerebellum and brainstem.	Real-time polymerase chain reaction	Pentamidine, fluconazole, flucytosine, and azithromycin	Deceased
27 (47)	Grand mal seizure	Exhibits a limited mass effect; reduced signal intensity on T1-weighted images, consistent with cerebral edema	Immunofluorescence assay	Treatment with anti-amoebic drugs	Not specified
28 (48)	Headache and nausea	There is a low-density area in the left frontal lobe.	Pathological testing	Azithromycin, flucytosine, rifampicin, and fluconazole	Deceased
29 (49)	Intermittent headaches	There is an expanding hypodense lesion in the right frontal lobe.	Indirect immunofluorescence staining	Fluconazole, trimethoprim–sulfamethoxazole, and rifampin	Deceased
30 (50)	High fever	Multiple areas of low density within the cranium, suggestive of multiple brain abscesses	Immunohistochemical testing	–	Deceased
31 (51)	Symptoms of somnolence	Multiple ring-enhancing lesions, some of which are accompanied by mild hemorrhage	Pathogen-specific polymerase chain reaction	–	Deceased
32 (52)	Weakness in the right leg	A low-density area in the motor region of the left frontal lobe	Pathological testing	Acyclovir, γ-globulin, and corticosteroids	Deceased
33 (53)	Recurrent fever	There are multiple abnormal signals in the cerebral parenchyma.	tNGS	Azithromycin, albendazole, trimethoprim–sulfamethoxazole, fluconazole, and fluorouracil	Deceased
34 (54)	Left-sided facial paralysis, speech difficulties, and weakness on the left side of the body	There is evidence of restricted diffusion in the right caudate nucleus, putamen, corona radiata, and the left putamen region.	Karius testing	Fluconazole, azithromycin, and trimethoprim-sulfamethoxazole	Deceased
35 (55)	Red patches and slightly hardened plaques on the bridge of the nose	There are multiple lesions throughout the brain, particularly in the frontal lobe.	Immunofluorescence staining assay	Pyrimethamine and fluconazole	Deceased
36 (56)	Sudden numbness and weakness in the left arm	There is an abnormal signal in the right parietal lobe.	mNGS	Trimethoprim-sulfamethoxazole, azithromycin, fluorouracil, and amphotericin B	Survived
37 (57)	Somnolence	Multiple lesions in the bilateral thalamus, midbrain, and bilateral cerebral white matter	Pathological testing	Steroid hormones	Deceased
38 (58)	Generalized tonic-clonic seizure	There is a ring-shaped hyperintense lesion in the left parietal lobe, surrounded by vasogenic edema.	Pathological testing	–	Deceased
39 (59)	Seizure symptoms	There is an area of low density in the left parietal lobe.	Molecular biology analysis	Amphotericin B, fluconazole, albendazole, doxycycline, rifampin, ethambutol, and meropenem	Deceased
40 (60)	Acute headache, difficulty walking, and impaired consciousness	Multiple lesions in the cerebral hemispheres, brainstem, and corpus callosum, accompanied by mild peripheral edema	mNGS	Antituberculosis and steroid therapy	Deceased
41 (61)	Intermittent nausea, vomiting, drowsiness, clumsiness, and weakness on the right side of the body	Multiple lesions have appeared in the left temporal and frontal lobes.	Pathological testing	Treatment with miltefosine and other anti-amoebic drugs	Deceased
42 (62)	Slurred speech, convulsions, and fever	There is a low-density lesion in the left frontal lobe.	mNGS	Miltefosine, fluconazole, rifampin, albendazole tablets, and amphotericin B	Not specified
43 (63)	Left-sided homonymous hemianopia	Extensive necrotic lesions are present in the right frontal lobe, right occipital lobe, and left parietal lobe.	NGS	Azithromycin, sulfamethoxazole, trimethoprim, and voriconazole	Deceased
44 (64)	Headaches and stomachaches	Multiple areas of abnormal signal are present in the bilateral lateral ventricles, the third ventricle, the fourth ventricle, the right temporal lobe, and the subventricular zone of the left occipital lobe.	mNGS	Penicillin and dexamethasone	Deceased
45 (65)	Drowsiness and irritability	Cerebral edema and cortical hemorrhage were observed in the right temporal, occipital, and posterior parietal lobes.	Pathological biopsy	Amphotericin B, isoniazid, rifampin, amikacin, moxifloxacin, and corticosteroids.	Deceased
46 (66)	Generalized seizures	There are lesions in both hemispheres of the brain	Pathological biopsy	Amphotericin, fluconazole, sulfamethoxazole-trimethoprim, meropenem, isoniazid, rifampin, ethambutol, pyrazinamide, and metronidazole	Deceased
47 (67)	Facial paralysis with drooping of the right side of the mouth	There are areas of decreased density in the right frontal lobe and the left frontoparietal lobe.	Pathological biopsy	Injections of immunoglobulin, methylprednisolone, and levetiracetam	Deceased
48 (68)	Confusion and rapid cognitive decline	Multiple lesions in the brain, accompanied by severe cerebral edema, meningeal thickening, and enhancement.	Pathological biopsy	Antibiotic treatment	Deceased
49 (69)	Seizure	There is extensive vascular edema in the left parietal and temporal lobes.	Indirect immunofluorescence assay	Broad-spectrum antibiotic therapy, phenytoin sodium therapy, and corticosteroid therapy	Deceased
50 (70)	Changes in vision and headaches	There are multiple lesions throughout the brain, accompanied by liquefaction of brain tissue and surrounding edema.	Pathological biopsy	Azithromycin, fluconazole, fluorouracil, and sulfadiazine	Deceased
51 (71)	Generalized seizures	There are multiple abnormal signals in the brain.	Pathological biopsy	Aminopterin, azithromycin, and miltefosine	Survived
52 (72)	An itchy rash has appeared on my right upper arm	There is a lesion in the posterior part of the right frontal lobe, with surrounding edema.	Pathological biopsy	Albendazole, fluconazole, and miltefosine	Deceased
53 (73)	Dizziness, vomiting, and blurred vision	There are hypodense lesions in the left lateral ventricle and the left occipital lobe.	mNGS	Albendazole, trimethoprim-sulfamethoxazole, and azithromycin	Deceased
54 (74)	A “small bump” has appeared on the right side of my nose that won’t heal and doesn’t hurt.	There are countless areas of focal or diffuse abnormal signals in both cerebral hemispheres, the corpus callosum, the brainstem, the cerebellum, and the spinal cord.	Pathological biopsy	Fluconazole and albendazole	Deceased
55 (75)	Repeated falls, worsening ataxia, irritability, and drowsiness	Multiple low-density lesions in the cerebral hemispheres	Pathological biopsy	Treatment with antimicrobial drugs such as cefuroxime and micafungin.	Deceased
56 (76)	Focal seizures	There is a mass in the cortical region of the left parietal lobe.	Pathological biopsy	Metronidazole, amphotericin B, and sulfadiazine/ethamidine	Deceased
57 (77)	Lump on the face	Abnormal signals in multiple areas of the brain	Pathological biopsy	Treatment for amoebiasis	Deceased
58 (78)	Headache, speech problems, and nervousness	Large space-occupying lesion in the brain	Pathological biopsy	Amphotericin B and miconazole	Deceased
59 (79)	A stinging sensation in the left temple and behind the left eye	T2 signal enhancement was observed in the left temporal lobe, accompanied by restricted diffusion, punctate hemorrhages, enhancement of the pia mater, and mild vascular edema.	Pathological biopsy	Vancomycin, imipenem, and acyclovir	Deceased
60 (80)	Back pain and headaches	There are large areas of abnormal lesions in the right temporal and frontal lobes, accompanied by surrounding vascular edema.	Pathological biopsy	Azithromycin, meropenem, metronidazole, fluconazole, trimethoprim-sulfamethoxazole, rifampin, trimethoprim-sulfamethoxazole, and amphotericin B	Deceased
61 (81)	Impaired consciousness and left-sided paralysis	High signal intensity is observed in the right insular cortex and the left occipital lobe.	Cerebrospinal fluid sediment microscope	Fluconazole, pentamidine isetionate, clarithromycin, albendazole, pentamidine, and fluorouracil	Survived
62 (82)	Forehead headache	There is an abnormal low-density signal in the cerebellar hemispheres.	Pathological biopsy	Rifampin, isoniazid, pyrazinamide, ethambutol, sulfadiazine, and pyrimethamine	Deceased
63 (83)	Progressive right-sided hemiplegia and speech difficulties	A low-density brain lesion is present in the left frontal lobe.	Pathological biopsy	Fluorouracil, fluconazole, pentamidine isethionate, sulfadiazine, and azithromycin	Survived
64 (83)	Generalized seizures, fever	There is edema in the left temporo-parietal region.	Pathological biopsy	Azithromycin, fluorouracil, fluconazole, sulindazole, and pentamidine	Survived
65 (84)	Pain in the back of the head, occasional confusion, blurred vision, and unsteady gait	Numerous peripheral hyperintense lesions are distributed throughout the brain, characterized by central liquefaction and surrounding edema.	Pathological biopsy	Fluorouracil, fluconazole, azithromycin, sulfadiazine, and miltefosine	Deceased
66 (85)	Paroxysmal epilepsy	There is a uniformly enhanced, isolated mass within the left frontal lobe.	Pathological biopsy	Glucocorticoid therapy	Deceased
67 (86)	Twitching in the right leg, neck spasms, numbness, headache, nausea, and flashes of light before the eyes	Multiple annularly enhanced lesions	Pathological biopsy	Pyrimethamine, sulfadiazine, fluorouracil, fluconazole, and azithromycin	Deceased
68 (87)	Sudden, severe headache and vomiting	Multiple ring-enhancing lesions	PCR testing	Phenylhydrazine, sulfadiazine, fluorouracil, fluconazole, azithromycin, and miltefosine	Survived
69 (87)	A firm, painless lesion appears in the middle of the face	Multiple lesions are present in both cerebral hemispheres, the brainstem, and the cerebellum	Pathological biopsy	Amphotericin B	Deceased
70 (6)	Double vision, slurred speech, and a low-grade fever	There is increased T2 signal intensity in the dorsal medulla, the medial portion of the left thalamus, and the right caudate nucleus; the right caudate nucleus is associated with a mild focal mass effect.	Immunofluorescence assay	Ceftriaxone, ampicillin, acyclovir, and vancomycin	Deceased
71 (88)	Headache and vomiting	Multiple poorly defined, heterogeneous intra-axial space-occupying lesions are present in the left cerebellum, bilateral frontal and parietal lobes, and the left temporal and occipital lobes.	PCR testing	Anti-tuberculosis and vancomycin	Deceased
72 (88)	Headache accompanied by double vision, fever, and vomiting	There are poorly defined lesions of varying sizes and signal intensities in the left temporo-parietal region, the left superior frontal-parietal region, the left parietal-occipital region, and the right superior parietal region. temporal and occipital lobes.	PCR testing	Anti-tuberculosis and vancomycin	Deceased
73 (88)	Headache accompanied by double vision, fever, and vomiting	There are poorly defined lesions of varying sizes and signal intensities in the left temporal region, the left parietal-occipital region, and the right side.	PCR testing	Albendazole, amphotericin, and clarithromycin	Deceased
74 (89)	A coin-sized, raised, red rash has appeared on the right side of the bridge of the nose, and the skin around the nostrils is bright red and eroded	There is a ring-shaped area of increased signal intensity within the right motor cortex.	PCR testing	Miltefosine, pentamidine, sulfadiazine, fluorouracil, fluconazole, and azithromycin	Deceased
75 (9)	Numbness on the right side of the face, left-sided hemiplegia, balance problems, and tinnitus	There is a lesion with enhanced margins in the right pons, accompanied by surrounding edema and mass effect.	mNGS	Cyclophosphamide	Deceased
76 (90)	Severe headache, blurred vision, and fever	There is an annular hyperintense lesion in the right parietal lobe, accompanied by edema and mass effect.	Immunofluorescence assay	Antiparasitic treatment	Deceased
77 (91)	Headache, fever	One non-enhancing, low-density lesion in the left caudate nucleus and one in the right parietal lobe	Fluorescent immunohistochemical analysis	Clocloxacillin, ceftriaxone, and amphotericin B	Deceased
78 (92)	Transient right-sided hemiplegia caused by epilepsy	A well-defined, uniformly enhancing space-occupying lesion is present in the subcortical region of the left frontal lobe, accompanied by perilesional edema and restricted diffusion.	Histopathological examination and polymerase chain reaction analysis	Glucocorticoid therapy	Deceased
79 (93)	Multiple skin lesions	Extensive, multifocal lesions caused by the mass effect	Pathological biopsy	Dexamethasone, azathioprine, rifampin, ketoconazole, metronidazole, ceftriaxone, and trimethoprim-sulfamethoxazole	Deceased
80 (94)	Headache	There is a lesion in the right medial frontal lobe, which shows patchy, annular enhancement accompanied by mild vasogenic edema.	Pathological biopsy	Itraconazole, fluorouracil, sulfadiazine, and azithromycin	Deceased
81 (95)	Granulomatous, noncaseating skin and soft tissue lesions of the face	There are multiple low-density lesions in the cerebral hemispheres.	Indirect immunofluorescence assay	Pyrimethamine, 5-fluorocytosine, and fluconazole	Deceased
82 (95)	Clonic seizures in the upper limbs and progressive loss of consciousness	There are multiple low-density lesions in the brain, primarily involving the bilateral frontal lobes.	Immunofluorescence assay	Phenylamine, 5-fluorocytosine, fluconazole, and clarithromycin	Deceased
83 (95)	Ulcerative skin lesions of the face	There are lesions in the left parietal and occipital lobes.	Immunofluorescence assay	Treatment with methyldopa, 5-fluorocytosine, fluconazole, and clarithromycin	Deceased
84 (96)	Fever, chills, persistent vomiting, and irritability	All ventricles are markedly dilated	Pathological testing	Isoniazid, rifampin, pyrazinamide, streptomycin and amphotericin B, and glucocorticoids	Deceased
85 (97)	Fever, focal seizures	There are multiple scattered areas of parenchymal enhancement in both cerebral hemispheres, surrounded by signal changes suggestive of edema.	PCR testing	–	Deceased
86 (98)	Chronic skin lesion on the right knee	Signal intensity in the left temporal lobe is increased, and a ring-shaped area of enhanced signal is visible within the temporal lobe.	Immunohistochemistry staining	Itraconazole and albendazole	Survived
87 (99)	Confusion, weakness on the left side of the body, and slurred speech	The lesion shows ring-shaped enhancement, accompanied by vascular edema around it.	Real-time PCR and conventional genotyping PCR tests	Rifampin, isoniazid, ethambutol, moxifloxacin, and prednisolone	Deceased
88 (100)	Impaired consciousness	Multiple areas of abnormally low density are present in both cerebral hemispheres.	PCR testing	Fluconazole, trimethoprim/ sulfamethoxazole, voriconazole	Deceased
89 (101)	A large, erythematous, purplish-red, erosive plaque has appeared on the back of the right hand	There is a solitary lesion in the right frontal lobe. The lesion appears as an irregular enhancing ring, accompanied by restricted peripheral diffusion and surrounding vascular edema.	PCR testing	Azithromycin, itraconazole, sulfadiazine, and fluorouracil	Survived
90 (102)	A tingling sensation in the right little finger and ring finger	There is a T2-hyperintense lesion in the right half of the medulla oblongata, with mild enhancement.	PCR, immunofluorescence	Amphotericin B and acyclovir	Deceased

In 2017, the U.S. Centers for Disease Control and Prevention (CDC) recommended a multidrug regimen comprising miltefosine, azithromycin, fluconazole, flucytosine, sulfadiazine, and other macrolide antibiotics for the treatment of granulomatous amebic encephalitis caused by *B. mandrillaris* (21, 22). Notably, in 2023, Spottiswoode et al. (23) described a 50-year-old patient with BAE who achieved a favorable clinical response to combination therapy with nitazoxanide, miltefosine, azithromycin, albendazole, and fluconazole. This case further supports the potential importance of early, aggressive multidrug therapy in the management of this frequently fatal infection. In China, one successfully treated case of BAE received a multidrug regimen comprising azithromycin, metronidazole, flucytosine, fluconazole, and trimethoprim–sulfamethoxazole (TMP–SMX) (24). Another successfully treated case reported in China received a broadly similar regimen, with the addition of sulfasalazine (25). Once the diagnosis was established, the patient was promptly initiated on antiparasitic therapy according to the CDC treatment guidelines. However, miltefosine, the recommended first-line agent, was not used due to its unavailability in the patient’s geographic region. Survival from BAE remains exceptionally rare. Both successful and unsuccessful cases reported previously have generally incorporated agents included in, or similar to, the multidrug regimens recommended by the U.S. CDC. In China, however, access to miltefosine may be limited. Consequently, alternative multidrug regimens consisting of agents such as azithromycin, metronidazole, flucytosine, fluconazole, and other potentially active antimicrobial agents have more frequently been used. Surgical intervention has also been performed in selected cases, particularly when focal or surgically accessible lesions were present. However, the efficacy of multidrug combination therapy and surgical intervention for BAE has not yet been established in well-designed clinical studies. Although a small number of survivors have been reported, the majority of treated patients have had poor outcomes. Previous reports have suggested that therapeutic failure may be related, at least in part, to limited drug penetration across the blood–brain barrier and the thick-walled cystic form of *B. mandrillaris*, which may reduce susceptibility to antimicrobial agents (24). We therefore speculate that treatment outcomes are likely influenced by multiple factors, including the timing of diagnosis, feasibility of surgical intervention, choice and combination of antimicrobial agents, and interindividual variability in disease severity and treatment response. Importantly, we do not consider the regimens used in fatal cases to be necessarily ineffective, as mortality may reflect the combined effects of advanced disease, pathogen burden, lesion location, host factors, drug availability, and other clinical variables. Further studies are needed to clarify the relative contribution of these factors and to define more effective therapeutic strategies for BAE.

## Conclusion

This case provides several important clinical implications. First, a negative mNGS result in CSF does not definitively exclude an infectious etiology. When an infectious disease is strongly suspected, the diagnosis should be based on an integrated assessment of the patient’s medical history, clinical manifestations, neuroimaging findings, and multiple laboratory investigations. When necessary, parallel mNGS testing of both serum and CSF, or repeated mNGS testing, may provide additional diagnostic evidence.

Second, this case highlights the complexity of the relationship between infection and secondary autoimmune responses. Infectious triggers may include not only viral pathogens but also other microorganisms, including parasites, and the causal interplay between infection and subsequent immune dysregulation may be multifaceted. Nevertheless, because our observations are derived from a single case, they are insufficient to establish a definitive causal relationship, which represents an important limitation of this report. We therefore propose only the possibility that parasitic infection may trigger or contribute to the development of autoimmune disease. Whether such a mechanism truly exists requires further investigation through experimental studies and larger clinical cohorts.

Despite these limitations, clinicians should be aware of the potential association between autoimmune encephalitis and *B. mandrillaris* infection. Greater awareness of this possible relationship may facilitate earlier recognition, improve diagnostic accuracy, and help identify the optimal therapeutic window, particularly in patients with atypical clinical presentations or inconclusive initial microbiological findings.

## Data Availability

The original contributions presented in this study are included in this article/supplementary material, further inquiries can be directed to the corresponding author.
